# Patellar socket technique for chronic quadriceps tendon repair: maximizing graft efficiency

**DOI:** 10.1007/s00402-025-06138-7

**Published:** 2025-11-27

**Authors:** Pasquale Porcelli, Kristijan Zoccola, Simone Cambursano, Riccardo Giai Via, Fortunato Giustra, Alessandro Massè, Marcello Capella

**Affiliations:** 1 Ospedale San Giovanni Bosco, Turin, Italy; 2 University of Turin, Turin, Italy; 3 CTO Hospital, Turin, Italy

**Keywords:** Chronic quadriceps tendon rupture, CQTRs, Quadriceps tendon repair, Autologous semitendinosus tendon, ST graft, Graft augmentation, Patellar socket

## Abstract

**Background:**

Chronic quadriceps tendon ruptures (CQTR) result in tendon retraction, fibrosis and tissue loss, often precluding direct repair. Traditional transosseous techniques require large length grafts and full-thickness patellar tunnels, increasing the risk of fractures.

**Objective:**

To present and evaluate a new surgical technique for reconstruction of CQTRs using an ipsilateral semitendinosus (ST) autograft fixed through a proximal patellar socket and Endobutton^®^ fixation, thus minimizing graft wastage and fracture risk.

**Methods:**

With the knee flexed at 90° and a thigh tourniquet, the ipsilateral ST is harvested through a medial incision, reinforced with FiberWire^®^-2 sutures, doubled and measured diametrically. A midline anterior approach exposed the chronic tendon defect, which must be debrided to the healthy margins. Three cavities are created in the proximal patella with an incannulated drill, each 5 mm laterally and medially and 7–8 mm centrally, only in the proximal part, to accommodate the thickness of the graft. The rest of the quadriceps is reinforced with FiberWire^®^-5 Krakow sutures. Using transport sutures, the prepared ST graft is inserted into the tendon via a Pulvertaft weft, inserted into the central cavity, stretched and secured over the distal patellar cortex with an Endobutton^®^. Krakow sutures for the native tendon and a FiberTape^®^ loop around the loop of the graft are passed through the respective tunnels and tied over the anterior patella. Intraoperative flexion confirms the stability of the construct; fluoroscopy verifies the height of the patella and the position of the Endobutton^®^.

**Conclusions:**

This proximal socket technique minimises the use of grafts and patellar stress, reliably restoring extensor mechanism function in CQTRs, offering a safe alternative to full transosseous tunnel methods.

## Introduction

Chronic quadriceps tendon ruptures (CQTRs) represent a significant clinical challenge as they severely compromise knee extension and the integrity of the extensor mechanism. These injuries typically occur when the quadriceps muscles undergo a sudden eccentric contraction, often as a protective response to prevent a fall during stair climbing or sports activities [1, 2]. In contrast to acute ruptures, in which the tendon extremities are fresh, minimally retracted and directly repairable with transosseous sutures or suture anchors [3], chronic ruptures are characterized by marked retraction, fibrosis and significant tissue loss (often more than 2 cm), which makes primary repair impractical [4, 5]. These features and previous repair failures or treatment delays beyond six weeks usually make graft augmentation necessary for successful reconstruction [4, 5].

Epidemiologically, these ruptures are generally unilateral and less common than their acute counterparts [6]. However, their incidence increases in older individuals, particularly in males between 50 and 70 years of age [6], and in patients with systemic diseases such as diabetes, obesity, chronic renal failure, rheumatoid arthritis, hyperparathyroidism and gout, as well as in those undergoing long-term corticosteroid or fluoroquinolone therapy [7–10]. Degenerative changes due to tendinopathy and cumulative microtrauma further contribute to the development of these chronic injuries. In some cases, chronic quadriceps tendon ruptures may also be iatrogenic due to unrecognized tendon injuries during total knee replacement surgery, excision of a tumor around the knee or fixation procedures [11, 12].

An accurate diagnosis is based on a complete clinical and radiological evaluation. Clinically, patients typically present with an inability to actively extend the knee, localized pain in the anterior aspect of the thigh and a palpable defect above the patella [1]. Radiographs may reveal indirect signs such as patella baja. At the same time, ultrasound (US) and magnetic resonance imaging (MRI) provide detailed information on the extent of tendon rupture, degree of retraction and tissue quality, which is crucial to distinguish between acute and chronic ruptures [13].

Direct repair with transosseous sutures or suture anchors offers excellent results for acute ruptures, as described in Literature [3]. In contrast, the retracted and fibrotic nature of chronic ruptures and the large gap between tendon ends precludes primary repair and makes graft augmentation necessary [4, 5]. Several graft options have been described, including autologous semitendinosus or gracilis tendons, long peroneus, iliotibial band autografts, local rotation flaps and Achilles tendon allografts [14–19]. However, many of these surgical techniques require the creation of a transverse patellar tunnel, which presents the risk of iatrogenic patellar fracture and consumes a larger volume of graft tissue, a disadvantage when dealing with extensive defects [20, 21].

In response to these challenges, we propose a new surgical technique to reconstruct chronic quadriceps tendon ruptures using an autologous ipsilateral semitendinosus tendon. This innovative surgical technique eliminates the need for a transverse patellar tunnel by creating a socket in the proximal portion of the patella with cannulated drills and tensioning the semitendinous tendon on an Endobutton^®^ (Smith & Nephew) distal to the patella. This reduces the risk of patellar fracture and preserves graft material to fill larger gaps effectively.

## Materials and methods

### Surgical technique

#### Patient positioning

The patient is positioned supine under regional anesthesia or general anesthesia with antibiotic prophylaxis administered according to the institutional protocol. A tourniquet is placed around the thigh. A bumper is placed to prevent excessive abduction during flexion and a roll is placed to position the knee at 90° of flexion.

The lower limb is then prepared and draped in a sterile field using disposable drapes. The lower limb is exsanguinated, and the thigh tourniquet is inflated to 280 mmHg.

#### Surgical technique

Anatomical Landmarks:

- Patella
- Patellar tendon
- Anterior tibial tuberosity
- Articular line
- Palpable defect at the quadriceps

## Semitendinosus tendon (ST) harvest and Preparation

**Fig. 1:**
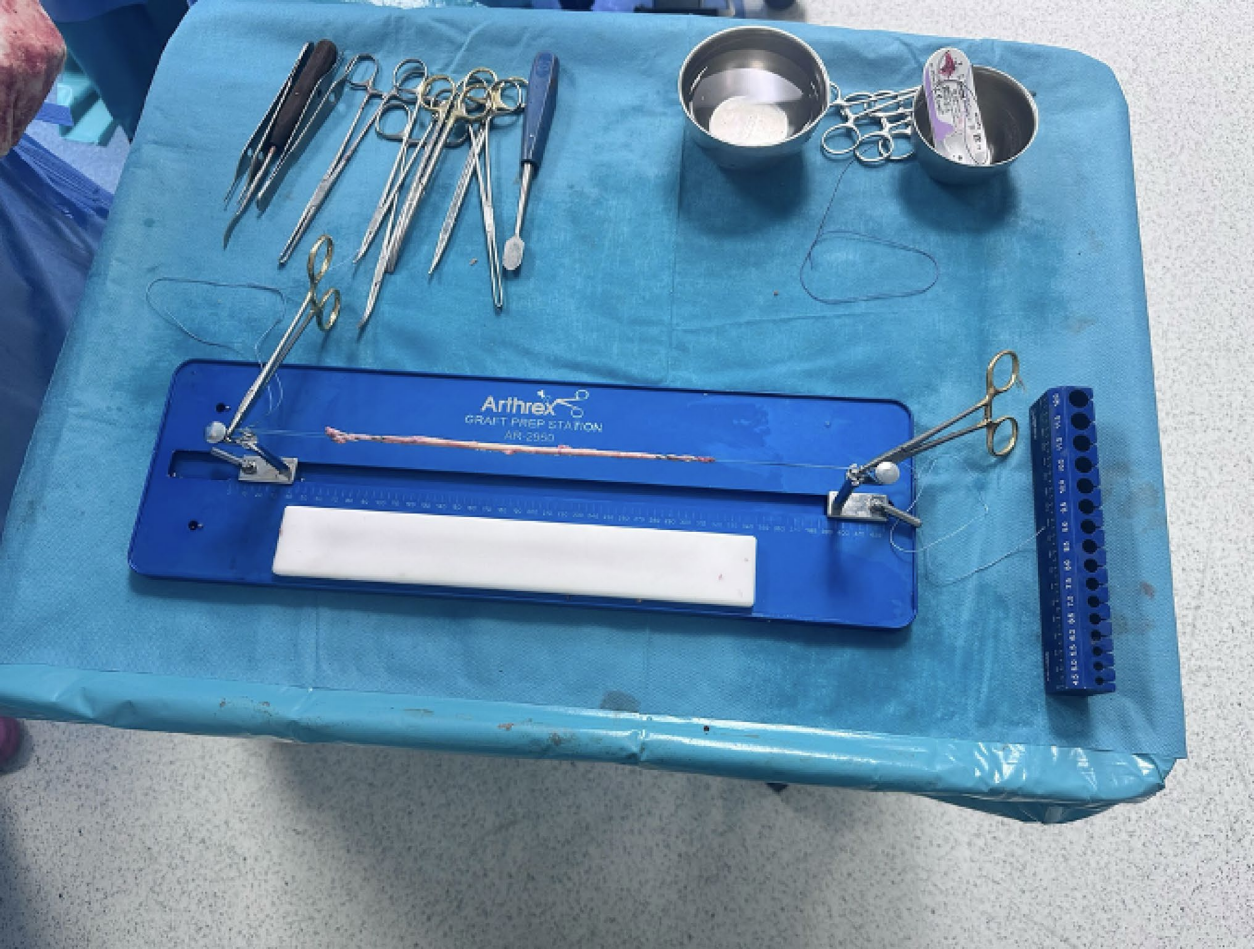
ST preparation on the workstation, at the ends of the graft the reinforcements with FiberWire^®^2 can be seen

A medial longitudinal incision was made three fingers distal to the joint line and two fingers medial to the tibial tuberosity to allow the ST to be identified and harvested with an open tendon stripper. The ST was then reinforced with FiberWire^®^ 2 (Arthrex) sutures on both ends to facilitate subsequent augmentation (see Fig. 1). The ST is duplicated and its diameter is then measured. This step is important because it gives an indication of the diameter of the socket that will be performed next in the proximal portion of the patella. The ST is then soaked in sterile saline solution with 500 mg Vancomycin dissolved in it.

## Quadriceps tendon exposure

A midline longitudinal incision is made over the anterior aspect of the knee. The soft tissues are dissected in layers to expose the quadriceps tendon and underlying patellar region. Dissection is performed along the tendon planes until the complete tear of the quadriceps tendon is identified; the poor tissue quality and gap are measured and noted. The tendon stump and patellar apex are carefully debrided to remove fibrotic tissue unsuitable for repair.

### Quadriceps tendon Preparation and augmentation

**Fig. 2:**
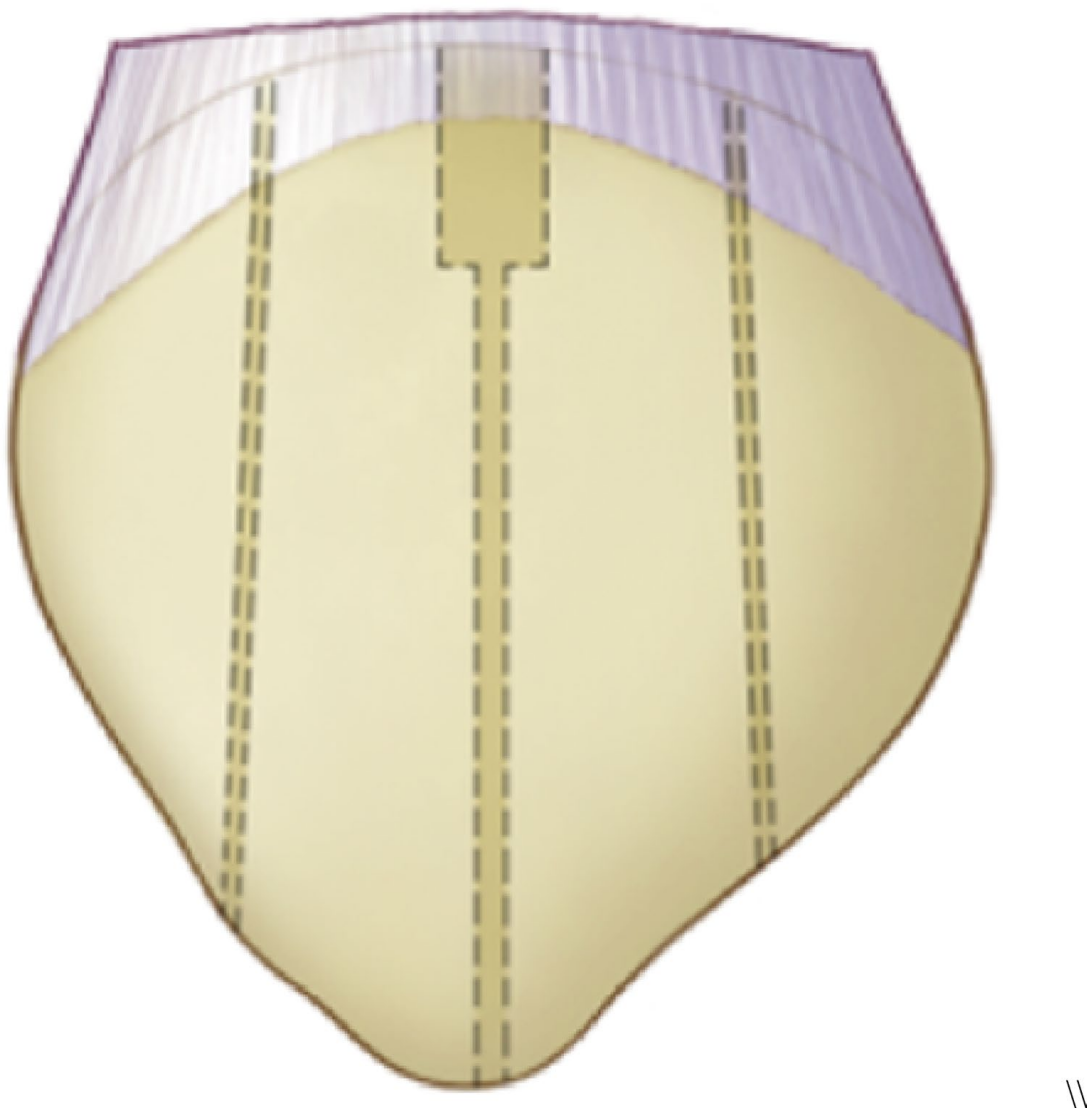
Stylized image showing the three vertical tunnels performed on the patella. You can also see that the central tunnel at the proximal level of the patella has a larger diameter where the bight of the ST graft will be accommodated

**Fig. 3:**
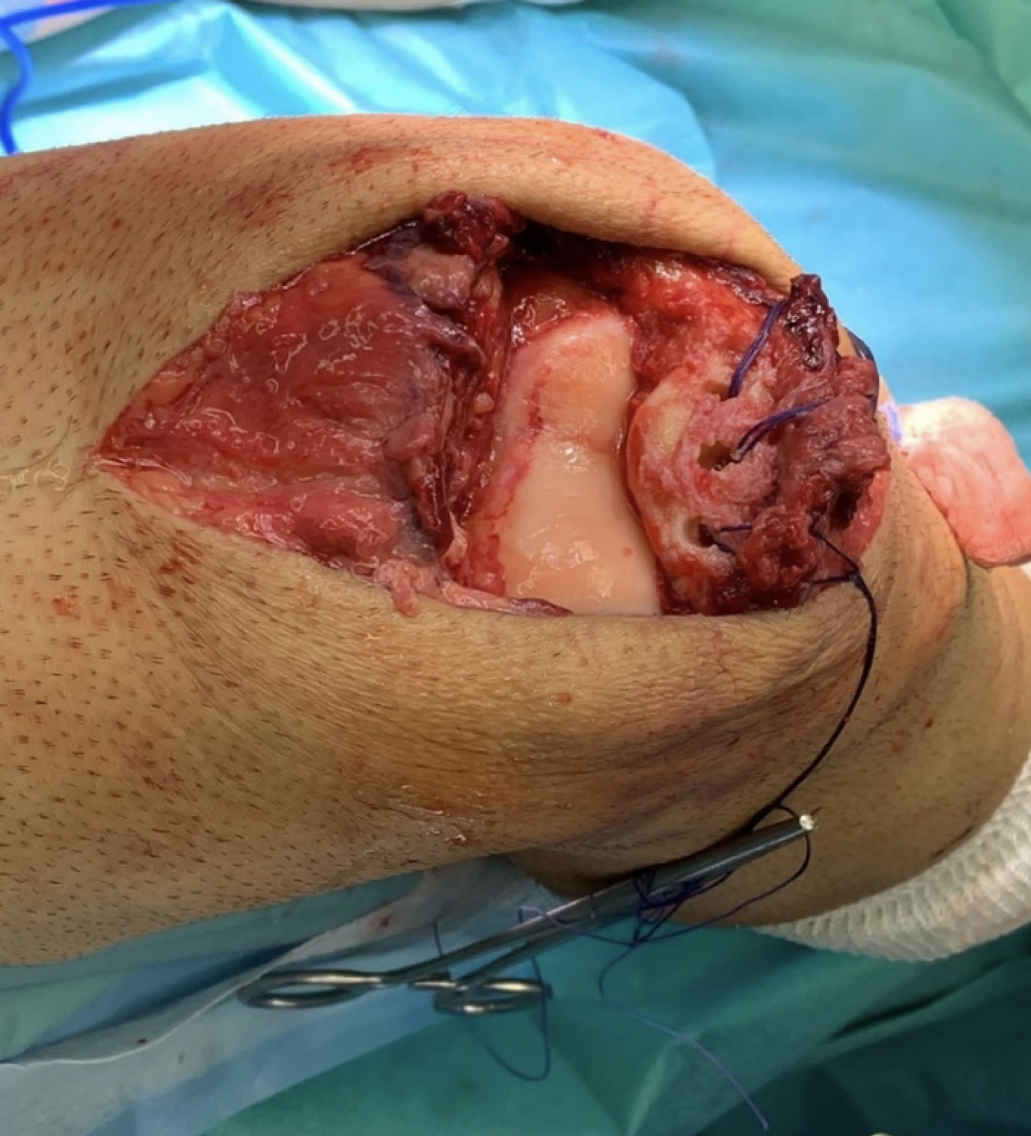
Intraoperative image of the three tunnels performed at the proximal patella level

The quadriceps tendon is then armed using two FiberWire^®^ 5 sutures using the Krakow technique, either on medial and lateral side of the tendon. Three tunnels are drilled through the patella as in the classic transosseous technique for acute quadriceps tenorrhaphy, using a “slotted” drill of 2.4 mm of diameter. Then using a cannulated drill, sockets are created for the ST graft, according to graft diameter which was previously measured. The lateral and medial socket around 5 mm, while the central one, that must embrace the bight of the ST is around 7–8 mm, according to the previous graft diameter measurement (see Figs. 2 and 3). The transport sutures are passed through these tunnels.

The ST graft is passed in augmentation at the level of the quadriceps tendon according to the Pulvertaft technique, thus creating a bight in the central portion of the ST.

**Fig. 4:**
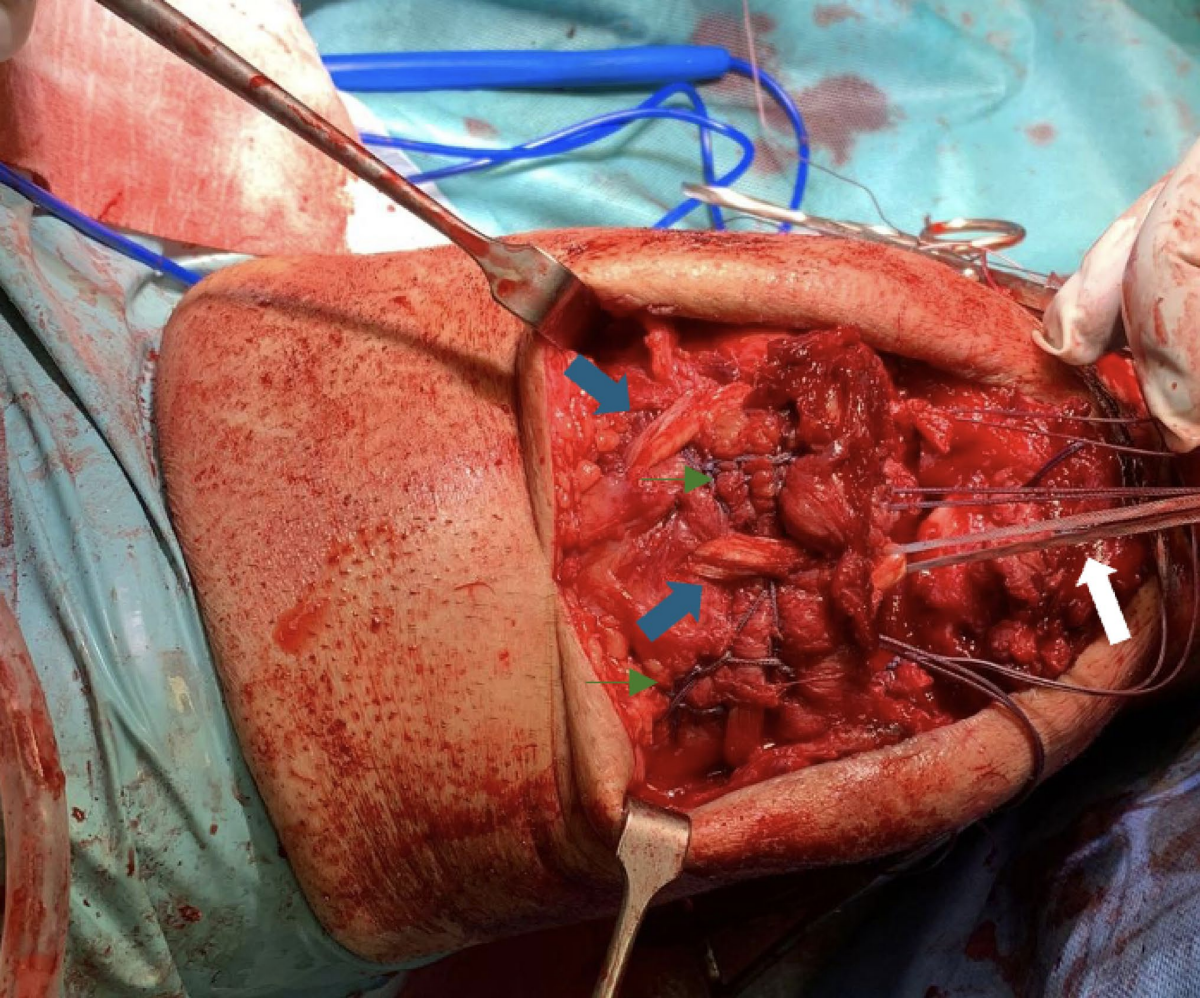
Intraoperative image in which the ST graft passed with the Pulvertaft technique inside the quadriceps can be seen, highlighted by the blue arrows; the reinforcement of the quadriceps with the Krakow suture technique is highlighted by the small green arrows. The white arrow points to the FiberTape^®^ placed around the loop of the ST

Finally one end of FiberWire^®^ 5 (armed quadricep tendon) and two ends of the FibeWire^®^ 2 (armed ST), on medial and lateral side, are passed through the medial and lateral tunnel. On central tunnel 4 ends are passed, both the medial and lateral remaining FiberWire^®^ 5 (armed quadricep tendon) and a FiberTape^®^ (Arthrex) around the bight of the ST (see Fig. 4).

**Fig. 5:**
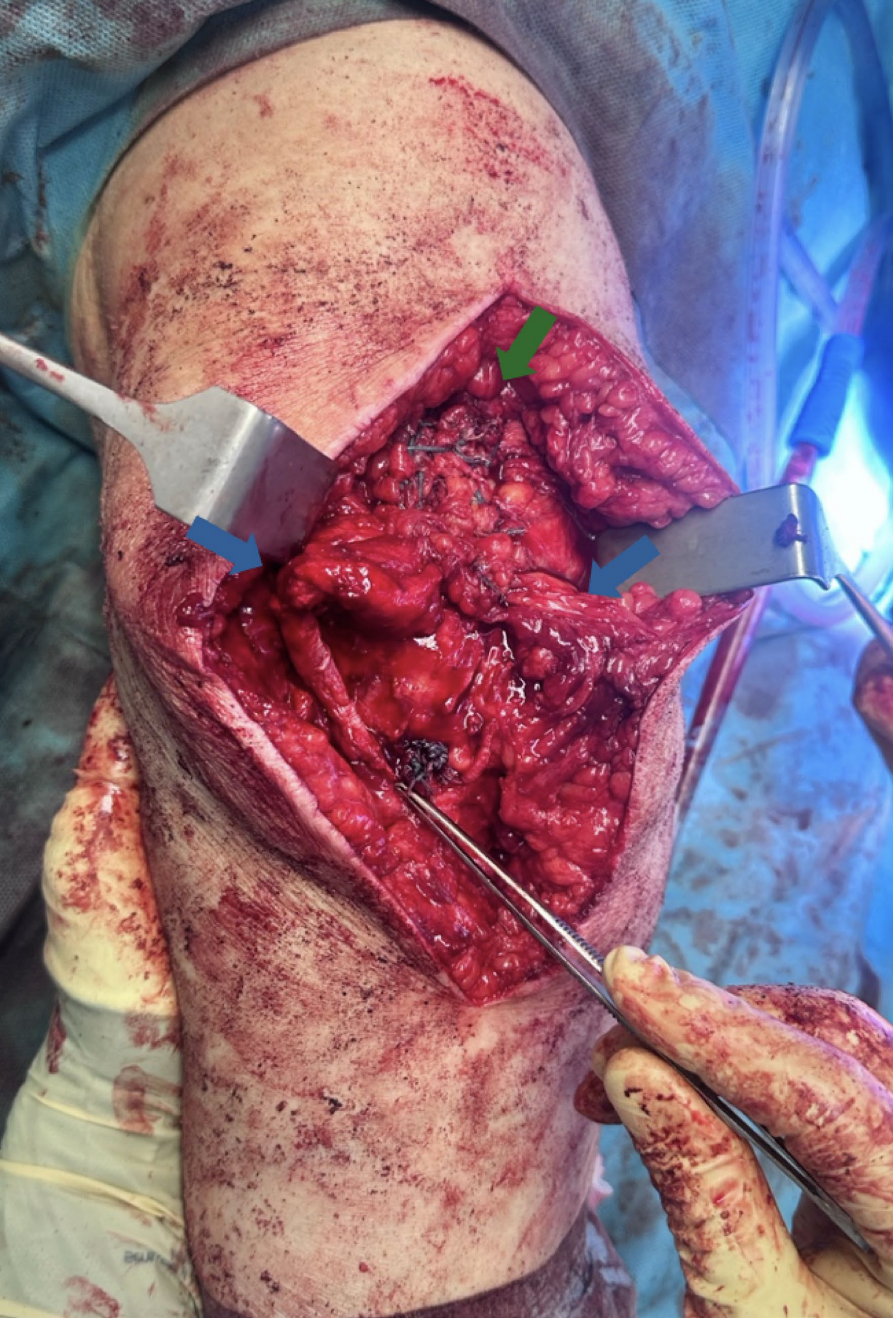
Final intraoperative image. The green arrow indicates the Krakow-type reinforcing suture at the level of the quadriceps, the blue arrows indicate the St used for augmentation. The surgical forceps indicate the distal pole of the patella where the Endobutton^®^ and the reinforcing Fiberwire^®^ nodes are located

The ST graft is stretched, indented into the proximal patella socket and fixed with an Endobutton^®^ placed on the distal aspect of the patella. To increase the tightness and to protect the ST, the quadriceps tendon is reinserted and fixed by anchoring the FiberWire^®^ sutures at the distal apex of the patella as in the classic transosseous technique (see Fig. 5).

## Stability check and wound closure

After completion, the stability of the reconstruction is checked, especially in flexion. The repair is reinforced with a continuous oversewing with a Vycril^®^ 2 (Ethicon). X-rays through the C-arm check the height of the patella and the correct positioning of the Endobutton^®^. Release of the tourniquet and accurate hemostasis. Layered wound closure is performed with absorbable sutures and a medical dressing.

Post-operative protocol:

- Non-weight-bearing protocol for 60 days.
- The knee brace is locked in extension for the first 20 days, followed by gradual unlocking (approximately 30° increase per week).
- Progressive weight-bearing beginning at 60 days post-surgery.
- Clinical and radiographic control at approximately 30-60-90 days post-surgery.

## Discussion

Chronic quadriceps tendon ruptures remain one of the most difficult injuries to deal with surgery due to tendon retraction, tissue degeneration and extensive scars. These injuries, typically defined as those occurring six weeks after injury, when the quality of the tendon is significantly compromised, cause severe impairment of the extensor mechanism. The integrity of the quadriceps tendon and the entire extensor apparatus is critical to allow extension movements, maintain ambulation, support jumping and sports activities, and ensure the normal function of the lower limb [22]. Although infrequent, chronic ruptures of the quadriceps tendon are extremely disabling injuries [23]. In addition, it is essential to consider that the anatomical location of the rupture can influence the surgical approach. Quadriceps tendon ruptures often occur at the tendon-bone junction or 1–2 cm from the superior pole of the patella, a hypovascularized region, which further complicates healing and repair [24].

Different techniques and case reports for the late treatment of quadriceps tendon ruptures have been described, reporting various surgical approaches with favorable outcomes [7, 14, 25, 26]. Indeed, in their review, Pengas et al. [27] identified multiple techniques for the delayed treatment of quadriceps tendon ruptures. These methods range from the traditional Codivilla technique [28] and the use of hamstring autografts to the application of synthetic materials, including autologous hamstring grafts, and even the use of a synthetic prolene tape (Ethicon) combined with platelet-rich plasma [29]. Recently, in their study, Elattar et al. [4] provided an overview of the management of chronic quadriceps tendon rupture and suggested a treatment algorithm based solely on the timing of diagnosis and surgery, noting that an early diagnosis can lead to better results and improved recovery of active and passive range of motion.

Recent approaches, such as the one by Oliva et al. [21], involve using an ipsilateral semitendinosus tendon graft passed through multiple transosseous tunnels in the patella arranged in a figure-of-eight configuration. This method requires a complete transverse drilling of the patella, which may predispose patients, especially those with osteoporotic or otherwise compromised bone, to an increased risk of iatrogenic fracture. Similarly, Rocha de Faria et al. [20] described a technique involving extensive debridement of tendon stumps followed by augmentation with a semitendinosus graft, and McCormick et al. [14] demonstrated that an autograft of semitendinosus and gracilis could be successfully used for revision chronic QTR to repair significant tendon defects.

Our modified technique differs from these methods by employing a proximal patellar socket instead of a full transverse tunnel. Using an Endobutton^®^ to tension the semitendinosus tendon, our approach reduces the amount of graft inserted into the patella to a minimum, thus preserving more graft, more bone structure, and significantly reducing the risk of patellar fracture. In essence, while classical techniques involve extensive passage of the graft through multiple patellar tunnels, our method simplifies the procedure, reduces the mechanical stress imposed on the patella, and allows more semitendinosus grafts to be available to fill the gap at the quadriceps level.

## Conclusions

The management of chronic quadriceps tendon ruptures remains challenging due to the lack of standardized, evidence-based guidelines. Our surgical technique uses an ipsilateral semitendinous tendon autograft, employing a proximal patellar socket approach that minimizes graft waste, reduces the risk of patella fracture and effectively reconstructs the quadriceps tendon.

## Data Availability

No datasets were generated or analysed during the current study.
